# Antioxidant and Antiapoptotic effect of aqueous extract of *Pueraria tuberosa* (Roxb. Ex Willd.) DC. On streptozotocin-induced diabetic nephropathy in rats

**DOI:** 10.1186/s12906-018-2221-x

**Published:** 2018-05-11

**Authors:** Rashmi Shukla, Somanshu Banerjee, Yamini B. Tripathi

**Affiliations:** 1 Department of Medicinal Chemistry, Institute of Medical Sciences, Banaras Hindu University, Varanasi, 221005 India; 2 Department of Zoology, Institute of Science, Banaras Hindu University, Varanasi, 221005 India

**Keywords:** *Pueraria tuberosa*, Diabetic nephropathy, Antioxidant, Antiapoptotic, Nephroprotective, GC-MS

## Abstract

**Background:**

Oxidative stress and renal apoptosis play a significant role in the progression of diabetic nephropathy. The tubers of *Pueraria tuberosa* (Roxb. ex Willd.) DC. has been traditionally used as anti-ageing and health promotive tonic. The purpose of this study was to investigate its nephroprotective effect and mechanism via antioxidant and antiapoptotic potential in Streptozotocin-induced diabetic nephropathy (DN) in rats.

**Methods:**

The chemical composition of aqueous extract of *Pueraria tuberosa* (PTY-2r) was analyzed by gas chromatography-mass spectrometry (GC-MS). Diabetes was induced by intraperitoneal injection of streptozotocin (STZ) (55 mg/kg body weight) in rats. After 60 days, the rats were randomly divided into 3 groups (*n* = 6/each group), namely DN control (DN) group-2, DN rats treated with PTY-2r at the dose of 50 mg/100 g, group-3 and 100 mg/100 g, group-4 p.o. for 20 days. The normal rats were chosen as a normal control (NC) group-1. PTY-2r was orally given to the rats for 20 days. Reactive oxygen species (ROS), lipid peroxidation (LPO) and the activity of ROS-scavenging enzymes – superoxide dismutase (SOD), catalase (CAT) & glutathione peroxidase (GPx) were determined in the kidney tissue of DN rats. The expression of apoptosis-related proteins was measured by immunofluorescence.

**Results:**

GC-MS analysis of PTY-2r indicated the presence of 37 compounds among them 5-Hydroxymethylfurfural (17.80%), 2,3-dihydro-3,5-dihydroxy-6-methyl-4H-pyran-4-one (17.03%), n-Hexadecanoic acid (5.18%) and 9-Octadecenoic acid (Z) - (6.69%) were found in the higher amount. A significant increase in ROS and LPO was observed along with the decreased activity of antioxidant enzymes, responsible for oxidative stress in the kidney of DN rats. Since, high oxidative stress induces apoptosis in target cells, as shown by significantly decreased expression of Bcl-2 along with increased expression of Bax, active Caspase-3 & cleaved PARP-1 in DN control rats, suggesting apoptosis. The PTY-2r treatment significantly raised the activity of antioxidant enzymes, suppressed oxidative stress and apoptosis thus, prevented urinary albumin excretion in a dose-dependent manner.

**Conclusions:**

The findings suggest that PTY-2r exerted the nephroprotective potential against STZ induced DN rats via suppressing oxidative stress and apoptosis due to the presence of different bioactive compounds.

**Graphical Abstract:**

ᅟ
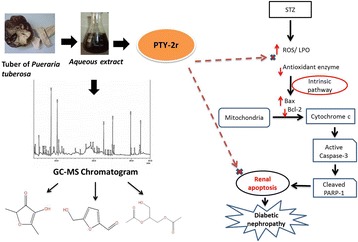

**Electronic supplementary material:**

The online version of this article (10.1186/s12906-018-2221-x) contains supplementary material, which is available to authorized users.

## Background

Diabetic nephropathy (DN) is one of the major complications of diabetes and the major leading cause of end-stage renal disease (ESRD). It is a progressive disease, assessed by a time-dependent rise in urinary albumin excretion and decline in renal functions [1]. In diabetic condition, hyperglycemia promotes oxidative stress and induced the excess generation of ROS, which plays a critical role in the pathogenesis of DN [2]. Increased production of ROS and oxidative stress (OS) causes enzyme inactivation, cell membrane damage, alteration in endogenous antioxidant gene expression and apoptosis [3]. In the kidney tissue, the high generation of ROS leads to renal apoptosis, resulting in glomerular barrier dysfunction and increased albumin excretion [4]. Renal diseases, particularly diabetic nephropathy has shown the features of apoptotic cell death [5–8]. Apoptosis may also cause podocyte loss in diabetic nephropathy and hence may lead to increased excretion of urinary albumin. These studies strongly suggested that ROS-mediated renal apoptosis is an early mechanism leading to DN. Thus, ROS-mediated renal apoptosis becomes a promising target for the development of nephroprotective drugs to prevent the initiation and progression of DN. The current therapeutics for DN is mainly antihypertensive drug such as angiotensin-converting enzyme (ACE) inhibitor and Angiotensin II receptor blockers (ARBs). Although for the treatment of DN, some drugs have been developed but they have not proven to be effective in clinical trials for clinical use [9]. Due to the limitation of allopathic drugs, the herbal medicines are being explored either as drugs or food supplements. The crude form of herbal medicines is a natural cocktail of phytochemicals and synergistically acts on different signaling pathways to get a better response [10, 11].

*Pueraria tuberosa* (PT) (Roxb. ex Willd.) DC. is a perennial herb commonly known as “‘vidarikanda” and distributed in the tropical parts of India [12]. The plant’s tuber is widely used in ethnomedicine as well as in traditional systems of medicine, particularly in Ayurveda. Its traditional clinical use in Ayurveda indicates its antiageing and health-promotive potential (named as “Rasayana drugs”) [13]. Its tubers are rich in flavonoids and isoflavones e.g., puerarin (8.31%), daidzein (1.70%) and genistein (1.37%), tuberosin,, 4-methoxypuerarin, quercetin, hydroxytuberosone, biochanin A, biochanin B, irisolidon, glycoside (C-glycoside 4′,6-diacetyl), puerarone, tectoridin and robinin [14, 15].

We have already reported its anti-inflammatory [16], antioxidant [17] and anti-diabetic properties [18, 19]. Studies also reported its anti-fertility [20], immunomodulatory and nootropic activity [21]. But not enough reports are available regarding its effect on chronic kidney disease like DN. In our earlier investigation, we reported that the aqueous extract of PT plays a beneficial role in reducing the STZ induced DN by inhibiting the accumulation of ECM via restoration of MMP-9 expression [18] and also effective in attenuation of hypoxia mediated DN by suppressing the expression of HIF-1α and VEGF [22]. In the current study, the bioactive constituents of PTY-2r were analyzed by GC-MS and elucidation of the mechanistic pathway through which PTY-2r ameliorates diabetic nephropathy in rats.

## Methods

### Chemicals

The PVDF membranes (catalog no. IPVH 20200, Millipore), mouse monoclonal β-actin (A2228) Horseradish peroxidase conjugated anti-rabbit IgG (A1949), STZ (S0130) were purchased from Sigma-Aldrich, St Louis, USA. Rabbit polyclonal Bcl-2 (N-19): SC492, rabbit polyclonal Bax (SC- 6236), were purchased from Santa Cruz Biotechnology, Inc. Cleaved PARP-1 ([E51], ab32064), rabbit anti-mouse IgG H&L (conjugated with FITC-green fluorescence, ab6724)and goat anti-rabbit IgG H&L (conjugated with TRITC-red, ab6718) were purchased from Abcam, USA. Active Caspase-3 (Asp175) purchased from Cell Signaling Technology, Inc. Prestained protein molecular weight marker was obtained from Hi-media Pvt. Ltd., Kolkata, India. All biochemical were of analytical grade. Biochemical kits were purchased from Accurex Biomedical Pvt. Ltd., Biosar, Thane.

### Extract preparation and gas chromatography- mass spectroscopy (GC-MS)

PT tubers were purchased from local market, authentication and extraction was done as described earlier [22]. In brief the tuber of *Pueraria tuberosa* (Roxb. ex Willd.) DC. was identified by Prof. K.N. Dwivedi, Department of DravyaGuna, Institute of Medical Science, Banaras Hindu University and also compared with the preserved sample in the herbarium of Department of Medicinal Chemistry Institute of Medical Science, Banaras Hindu University, (voucher no. YBT/MC/12/1–2007). 50 g of coarse powder of PT was boiled with 5volume of water. Volume reduced to ¼^th^ and filtered. Filtered extract was washed with hexane in separating funnel then aqueous part (PTY-2r) was collected and concentrated by a rotatory evaporator and lyophilized. It stored at − 20 °C until use. The chemical compositions of PTY-2r were analyzed by GC-MS.

GC-MS analysis was carried out by using Shimadzu QP-2010 Plus with Thermal Desorption System TD 20 with quadrupole detector; the injection temperature was 260 °C in split mode. The pressure was 77.5 kPa. The oven temperature was initially kept at 70 °C with holds time of 2.00 min, and then raised to 250 °C at the rate of 7 °C /2 min then 280 °C at the rate of 10 °C with hold time of 28.00 min. The ion source and interface temperature was 230 °C and 270 °C respectively. The compounds were identified by comparing mass spectra with those of NIST (WILLEY8.LIB) mass spectral library.

### Preliminary quantitative chemical analysis

Total phenolic content was estimated by folin-ciocalteu assay [23] and expressed in terms of μg gallic acid equivalent (GAE/mg). Gallic acid was used as a standard solution. Briefly, 0.5 ml of sample was mixed with 1.0 mL of 1 N of Folin–Ciocalteu reagent. The contents were mixed and allowed to stand for 5 min at room temperature. Next, 1 mL of 75% sodium carbonate solution was added, followed by distilled water. Solutions were mixed and allowed to stand at room temperature for 15 min, and then absorbance was recorded at 760 nm against distil water as blank.

Total flavonoids content was measured by using aluminum chloride (2%) [24] in which it was mixed with solution of test samples. Absorbance measured at 415 nm (Elico SL177) after 10 min against a blank sample consisting of 1.0 mL of sample solution and 1.0 ml of methanol without aluminum chloride. The total flavonoids content was determined by using a standard curve of quercetin at 0–150 μg/mL. The average of three readings were used and then expressed in μg of quercetin equivalent to flavones per mg extract.

Tannin content was estimated as described by Van Baren [25] with slight modification. Tannic acid used as standard solution. 1 mL of extract was mixed with 2 ml of 0.1 M FeCl3 in 0.1 N HCl and 0.008 M potassium ferrocyanide. The absorbance was measured at 720 nm within 10 min. Using spectrophotometer (Elico SL177). Tannin content was expressed in terms of μg tannic acid equivalent (TAE) /mg.

### Animal experiment detail and induction of DN

The male rats of Charles Foster strain (100-120 g) were injected with STZ (55 mg/kg body weight, freshly prepared in citrate buffer of pH 4.5, *i.p.*) after the overnight fasting. The rats with a blood glucose level higher than 17 mmol/l were considered as diabetic [26]. DN was induced by maintaining the persistent hyperglycemia for 60 days in rats. The periodic checks of renal function tests, diabetes and urine albumin was done upto 60 days after every 10 days. The rats with positive albuminuria were considered as diabetic nephropathy bearing rats and they were randomly divided into different groups.

### Treatment protocol

The normal rats were chosen as a normal control (NC, *n* = 6, group-1), STZ induced DN control rats, (DN, n = 6, group-2), STZ induced DN rats, treated with PTY-2r (DN + PTY-2r, 50 mg/100 g body weight, n = 6, group-3) & STZ induced DN rats, treated with PTY-2r (DN + PTY-2r, 100 mg/100 g body weight, n = 6, group-4). The rats of group-1 and group-2 were given drug vector. After 20 days of treatment, all the rats were sacrificed by giving anesthesia with 1% pentobarbital (i.p. 45 mg/kg body weight) and the left kidneys were snap-frozen in liquid nitrogen and stored at − 80 **°**C for biochemical estimation. The right kidneys were fixed in 10% neutral formalin for immunofluorescence studies. The experiment was conducted in accordance with institutional practice and within the framework of the revised Animals (Scientific procedures) Act of 2002 of the Government of India on Animal Welfare.

### Urinary albumin excretion (UAE)

Rats were placed into the metabolic cages (Vishnu traders, Roorkee-247,667, Uttrakhand, India) for 24 h urine collections at the end of the experiment. A pinch of thymol was added to the urine collection beaker to prevent the microbial growth. Estimation of UAE was done byAfinion™AS100 Analyzer in Parul Pathology, Lanka Varanasi, India.

### Total ROS level

The total ROS generation in the fresh homogenate of tissue was assessed by using the method of Bejma et al. 2000 [27] with slight modification [28]. Briefly, the homogenate was diluted in PBS to obtain a concentration of 25 μg tissue protein/ml. The reaction mixture containing diluted homogenate and 10 μl of 2′ 7′- dichloro dihydro fluorescein diacetate (DCFH-DA) (10 μM) was incubated for 15 min at room temperature. After 30 min of further incubation, the conversion of DCFH-DA to the fluorescent product DCF was measured by using a spectrofluorometer with excitation at 484 nm and emission at 530 nm.

### Lipid peroxidation assay (LPO)

The level of lipid peroxides was measured as thiobarbituric acid reacting substance (TBARS) and expressed as equivalent to malondialdehyde (MDA) by using 1,1,3,3-tetramethoxypropane (TEP) as standard in kidney homogenate [29]. It was expressed in terms of μmol/mg protein.

### Antioxidant enzyme activity in kidney tissue

The kidney tissue homogenate (10% *w*/*v*), was prepared in 0.1 M potassium phosphate buffer (pH 7) containing a protease inhibitor. It was centrifuged at 10,000 g for 20 min at 4 °C and the clear supernatant was utilized for assay of superoxide dismutase (SOD) [30], Catalase (CAT) [31], glutathione peroxidase (GPx) [32].

#### Superoxide dismutase (SOD)

Activity was determined in terms of inhibition of reduction of nitro blue tetrazolium (NBT) in the presence of riboflavin as described [30] with slight modification [33]. In brief, each 3 ml of reaction mixture contained 0.01 M phosphate buffer (PBS, pH 7.8), 130 mM methionine, 0.5 mM EDTA, 0.75 mM NBT, 60 μM riboflavin and 0.5 ml of kidney homogenate. It was kept in front of a fluorescent light and after 6 min absorbance was taken at 560 nm. The SOD activity was expressed as U/mg protein.

#### Catalase activity (CAT)

Was assessed by Aebi’s method [31] by measuring the rate of decomposition of H_2_O_2_ at 240 nm as reported earlier [34]. In brief, the reaction mixture contained 1.9 ml of 50 mM PBS (pH 7.0) and diluted kidney homogenate to make volume 2 ml. The reaction was initiated by the addition of 30 mM hydrogen peroxide (H_2_O_2_) and absorbance was measured at 240 nm for 2–3 min. The catalase activity expressed in terms of U/mg protein.

#### Glutathion peroxidase (GPx) activity

Was assayed as described by Mantha et al. [32]. In brief, 50 μl of sample was added to a reaction mix containing 398 μl of 50 mM phosphate buffer, 2 μl of 1 mM EDTA, 10 μl of 1 mM sodium azide, 500 μl of 0.5 mM NADPH, 40 μl of 0.2 mM GSH and 1 U glutathione reductase and allowed to equilibrate at room temperature. Then, the reaction was initiated by addition of 100 mM H_2_O_2_. The absorbance measured kinetically at 340 nm. The GPx activity was expressed as μmol of oxidized NADPH oxidized to NADP^+^ per min per mg of protein.

### Immunofluorescence for Bax, Bcl-2, active Caspase-3, & cleaved PARP-1 in the kidney sections using confocal microscopy

The kidney tissue was fixed in 10% formalin, embedded in paraffin wax and 5 μm thick sections were cut by using the rotatory microtome, Lieca RM2125 RT (Leica Biosystems Nussloch GmbH, Nussloch, Germany) and the Immunofluorescence of Bax, Bcl-2, active Caspase-3 and cleaved PARP-1 was performed in a two-step procedure as mentioned elsewhere [35]. Briefly, kidney sections were deparaffinized in xylene and rehydrated in graded series of alcohol. Heat induced antigen retrieval was performed using 1 M citrate buffer (pH 6) in a microwave oven at 1000 W (1–2 min). After treating with blocking solution for 2 h at room temperature (5% HIGS- Heat Inactivated Goat serum), different sections were incubated with different antibodies Bax(dilution1:25), Bcl-2(dilution 1:50), active Caspase-3 (dilution 1:50) and cleaved PARP-1 (dilution 1:20) for 24 h in humid chamber. Sections were then washed with TBS (3 X 5 min.) and incubated with rabbit anti-mouse IgG H&L (conjugated with FITC) and goat anti-rabbit IgG H&L (conjugated with TRITC), (1:200) for 3 h. at room temperature in dark. After incubation, sections were again washed with TBST(tris bufferes saline with tween-20) (3 X 5 min.) and two drops of the fluorescent media (0.5% N-propyl gallate + 20 mM Tris in 90% glycerol + DAPI) were applied on the sections. Coverslips were applied and then sealed with nail polish after ensuring the spread of the mounting media over all the sections without any bubble formation). Nuclei were counterstained with DAPI (1 μg/10 ml PBS).

### Image analysis

Immunofluorescence of Bcl-2, Bax, active Caspase-3 and cleaved PARP-1 in the kidney section of all the groups were observed under a Zeiss LSM510 Meta laser-scanning confocal microscope with a Plan-Apo 20.0×, 1.4- NA oil immersion objective and the images were collected using LSM 510 Meta software in same magnification and stored in TIFF (tag index figure format) files. Integrated optical density (IOD) of all images were measured and analyzed using Image J software (Image J 1.48, National Institute of Health, Bethesda, MA, USA) and signal intensity of immune-positive signals were measured as mentioned previously [35]. The IOD values (arbitrary unit) were averaged (four sections per kidney and 6 kidneys per group) to determine the signal density. Based on the signal intensity, the term intense, moderate and weak were applied.

### Statistical analysis

All data were presented as the mean ± SD. One-way ANOVA followed by post-Hoc analysis (Dunnett’s T3) was used to determine the significance among different groups. A *p*-value of < 0.05 was considered as significant and data was analyzed using the statistical software package, Statistical Analysis System (SPSS Statistics 20.0, IBM, Armonk, NY, USA).

## Results

### Preliminary quantitation of chemical constituents in PTY-2r

Preliminary phytochemical analysis revealed that the PTY-2r rich in total phenols, flavonoids and tannins, as a major constituents as shown in Table 1. Total phenolic content was found to be 150 ± 9.56 μg/mg of Gallic acid equivalent and total tannin i.e. polyphenolic content was observed to be 72 ± 3.54 μg/mg of tannic acid equivalent. Total flavonoid content was estimated15.9 ± 2.67 μg/mg of quercetin equivalent.

**Table 1:** Quantitative estimation of phytochemicals present in PTY-2r

Fraction of PT	Total phenolic content (μg of GAE/mg)	Total flavone content (μg of QE/ mg)	Total tannin content (μg of TAE/ mg)
PTY-2r	150 ± 9.56	72 ± 3.54	5.9 ± 2.67

Values are means of three independent determinations ± standard deviation (SD)

### GC-MS analysis

**Fig. 1:**
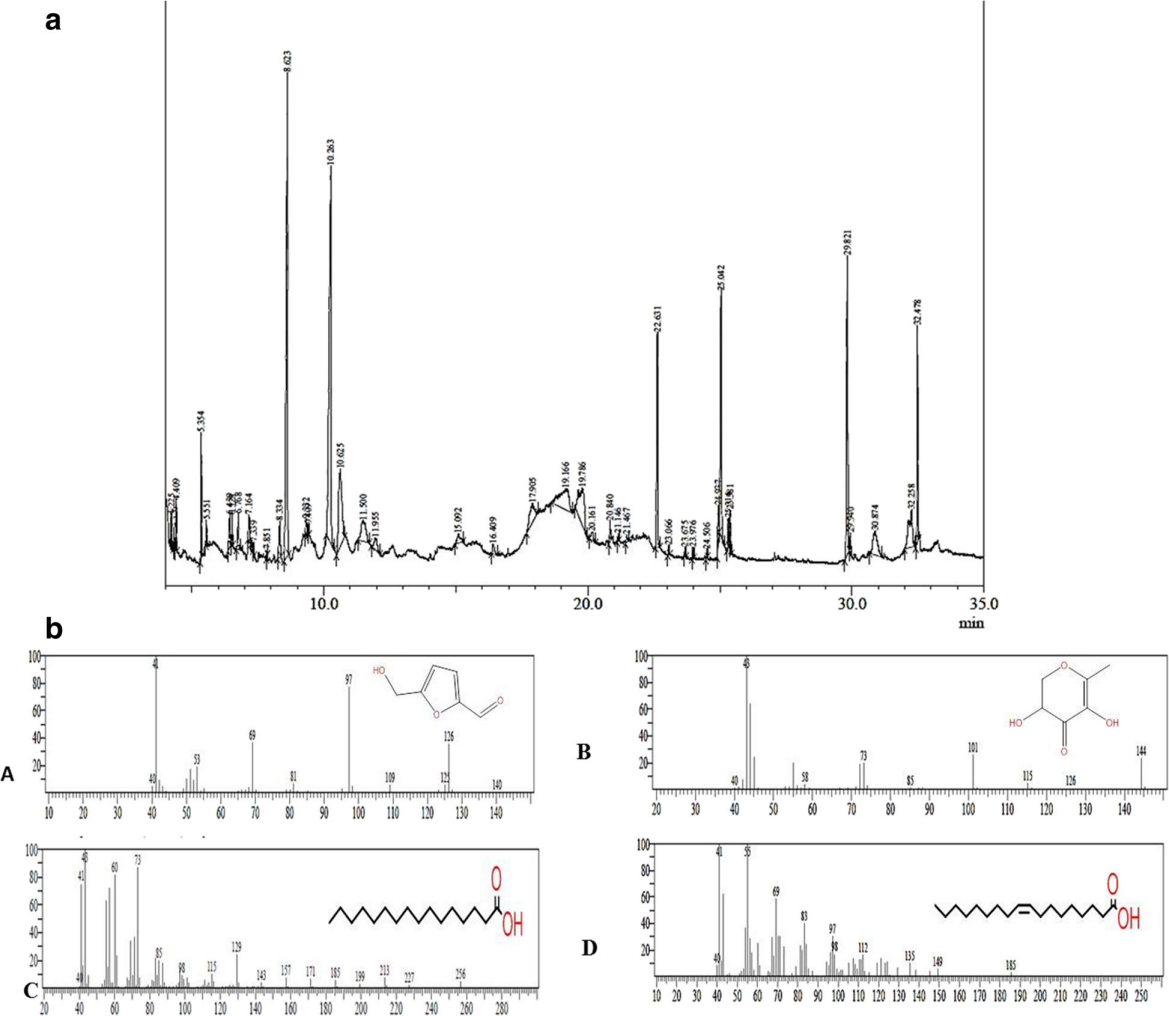
**a**.GC-MS Chromatogram of PTY-2r. **b** Spectra of major compounds (**A**) Spectra of 2, 3-dihydro-3, 5-dihydroxy-6-methyl-4H-pyran-4-one, (**B**) Spectra of 5-Hydroxymethylfurfural, (**C**) Spectra of n-Hexadecanoic acid (**D**) Spectra of 9-Octadecenoic acid (Z)-

Total 37compounds were identified in the PTY-2r are shown in Additional file 1: Table S1 along with their structure and reported activities. The major constituents present in the extract were 5-Hydroxymethylfurfural (17.80%) which is reported as antiapoptotic, 2, 3-dihydro-3, 5-dihydroxy-6-methyl-4H-pyran-4-one (17.08%) reported as strong antioxidant, Other minor constituents are 9-Octadecenoic acid (Z) (6.69%) and n-Hexadecanoic acid (5.18%) are also reported as antioxidant, as shown in Table 2 with their reported activity. Fig. 1 shows the GC-MS chromatogram and the spectra of major compounds.

**Table 2:** Major compounds identified in PTY-2r through GC-MS analysis

S.no	R.time	Name	Area	Area%	Formula	Molar mass (g/mol)	Structure	Reported activity
1.	8.623	2,3-dihydro-3,5-dihydroxy-6-methyl-4H-pyran-4-one	23,642,509	17.03	C_6_H_8_O_4_	144.126		Antioxidant, antifungal [41]. anti-inflammatory, anti-proliferative & pro-apoptotic [56]
2.	10.263	5-Hydroxymethylfurfural	24,722,467	17.80	C_6_H_6_O_3_	126.111		Anti-apoptotic [51, 52]
3.	22.631	n-Hexadecanoic acid	7,188,212	5.18	C_16_H_32_O_2_	256.43		Antioxidant and Bactericidal activity [57, 58], Anti-inflammatory, antimicrobial [59].
4.	23.675	9-Octadecenoic acid (Z)-	201,065	6.69	C_14_H_26_O_2_	226.36		Anticancer, Anti-inflammatory, 5-alpha reductase inhibitor [58, 60]

R.time- Retention time

### Effect of PTY-2r the overproduction of free radicals

**Fig. 2:**
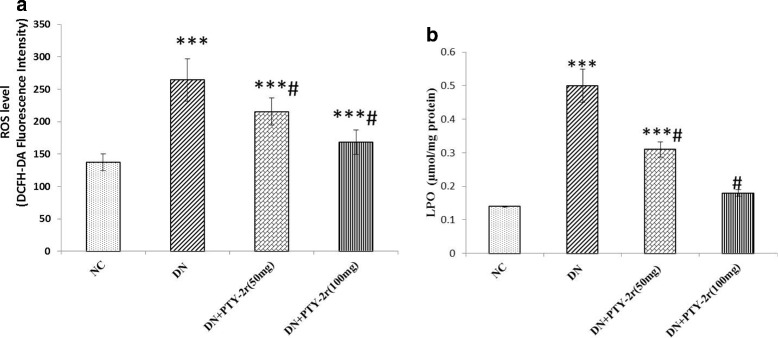
Effects of PTY-2r on Overproduction of Free Radicals: (**a**) ROS measured by DCFH-DA fluorescence intensity (**b**) Concentration of LPO in the kidney of NC, DN control, DN + PTY-2r (50 mg/100 g) and DN + PTY-2r (100 mg/100 g). Data are presented in mean ± SD (*n* = 6 in each group). ***p < 0.001, compared with group-1, #*p* < 0.001 compared with group-2

In the renal tissue of DN rats, high oxidative stress was observed, as ROS & LPO content were significantly increased, when compared to NC rats (*p* < 0.001). However, the PTY-2r treatment significantly reversed these changes and decreased the ROS production in dose-dependent manner (Fig. 2a). Overproduction of ROS was further supported by the high degree of lipid peroxides, which was measured as thiobarbituric reactive substances (TBARS). The malondialdehyde (MDA) is the main product of LPO, which increases during oxidative stress, MDA level was significantly increased in DN control rats compared to NC group, while PTY-2r treatment significantly decreased its concentration in dose-dependent manner (Fig. 2b). The higher dose (100 mg) was more effective than low dose (50 mg).

### Effect of PTY-2r on the activity of antioxidant enzymes

**Fig. 3:**
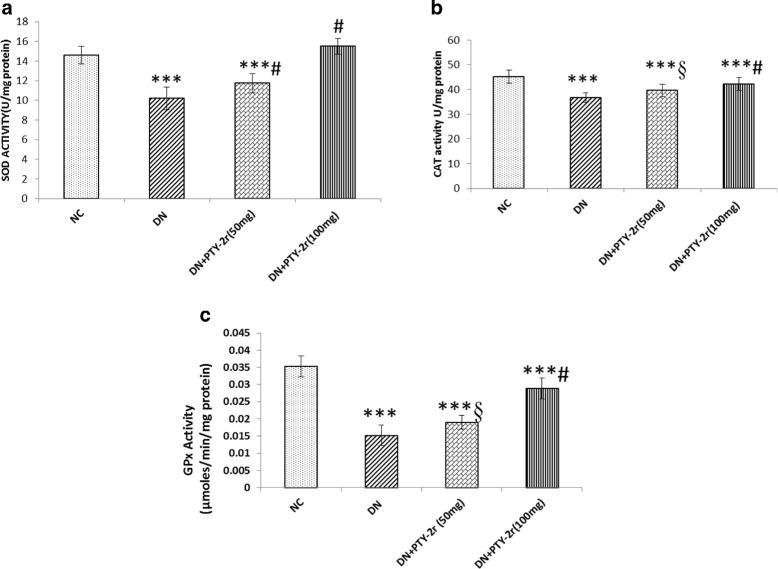
Effects of PTY-2r on Activity of Antioxidant Enzymes through Biochemical Estimation: (**a**) SOD (**b**) CAT (**c**) GPx in NC, DN control, DN + PTY-2r (50 mg/100 g) and DN + PTY-2r (100 mg/100 g). Data are presented in mean ± SD (*n* = 6 in each group). ***p < 0.001 compared with group-1; ^#^*p* < 0.001, ^§^*p* < 0.05, compared with group-2

Since, there was increased production of ROS which suppressed the activity of antioxidant enzymes, so we further checked the activity of antioxidant enzymes (SOD, CAT &GPx) in kidney tissue of all the groups. As shown in Fig. 3, the activity of the antioxidant enzymes was significantly decreased in DN rats compared to NC, while 20 days of PTY-2r treatment significantly restores the activity of these enzymes in a dose-dependent manner but the effect was more significant in higher dose.

### Effect of PTY-2r on renal apoptosis by using immunofluorescence

**Fig. 4:**
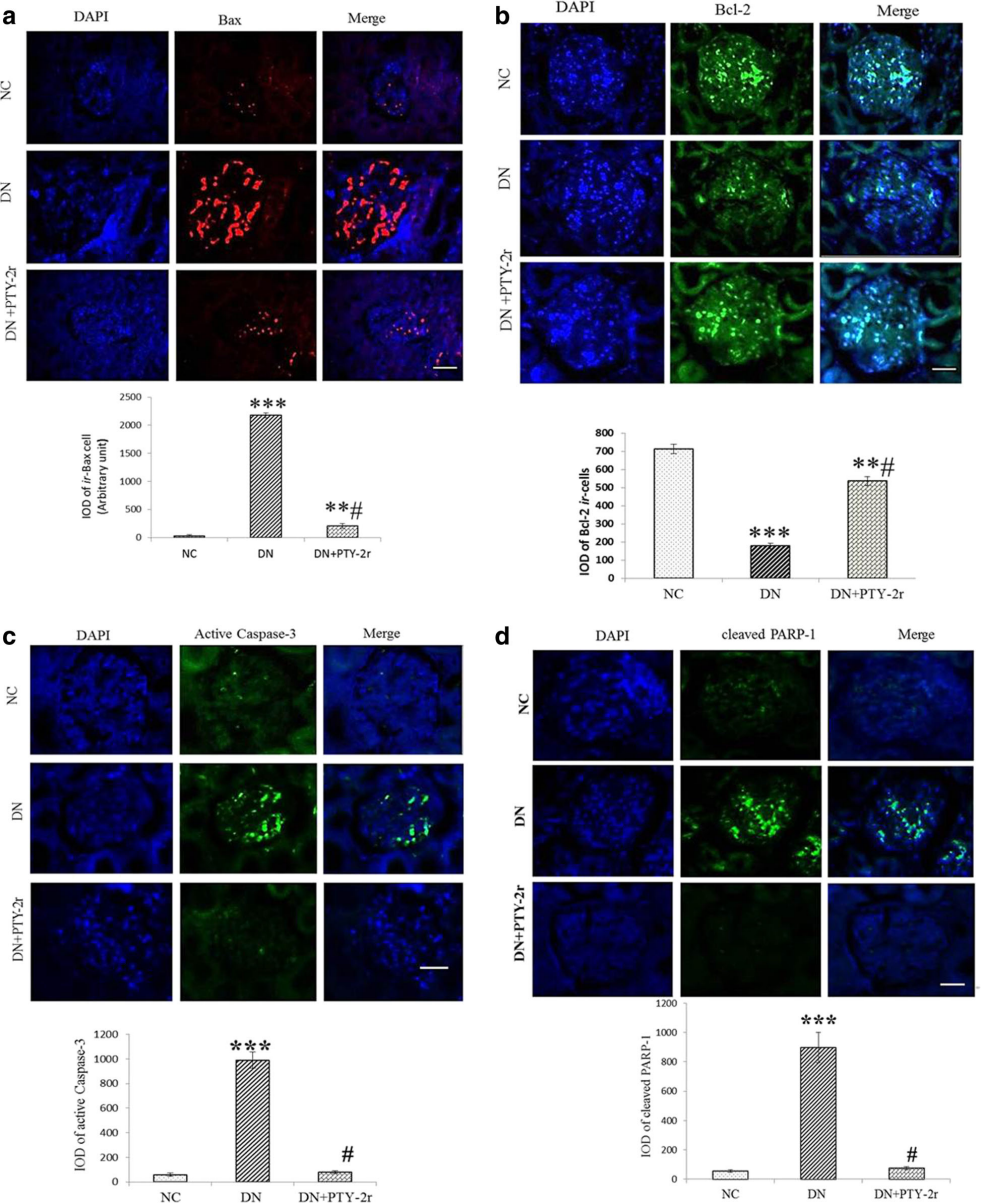
Effects of PTY-2r on Renal Apoptosis: (**a**) Immunofluorescence of Bax in NC, DN rats, and DN + PTY-2r (100 mg/100 g) rats and integrated optical density (IOD) of *ir*- Bax cells. (**b**) Immunofluorescence of Bcl-2 in NC, DN rats, and DN + PTY-2r (100 mg/100 g) rats and integrated optical density (IOD) (**c**) Immunofluorescence of active Caspase-3 in NC, DN rats, and DN + PTY-2r (100 mg/100 g) rats and integrated optical density (IOD). **d** Immunofluorescence of cleaved PARP-1 in NC, DN rats, and DN + PTY-2r (100 mg/100 g) rats and integrated optical density (IOD). Scale bar, 50 μm. All data presented in mean ± SD, (*n* = 6 in each group). ***p < 0.001, **p < 0.05 compared with group-1; ^#^*p* < 0.001 compared with group-2

As the generation of ROS is responsible for renal apoptosis, thus we further examined the expression of antiapoptotic protein i.e. Bcl-2 & pro-apoptotic i.e. Bax, active Caspase-3 and cleaved PARP-1 in the kidney tissue of all the groups. Biochemical analysis showed that the 100 mg/100 g dose of PTY-2r is more efficient, thus we further checked the effect of PTY-2r (100 mg/100 g) on immunoreactivity of apoptotic proteins in the kidney of DN rats by immunofluorescence. As shown by immunofluorescence of kidney sections (Fig. 4-a, b, c & d), the DN rats showed significantly intense immunoreactivity of Bax (2174.16 ± 385.499, *p* < 0.001), active Caspase-3 (1527.2 ± 83.2, p < 0.001) & cleaved PARP-1 (2144.5 ± 212.04, p < 0.001) while weak immunoreactivity was found for Bcl-2 (177.9 ± 30.7, p < 0.001) in glomerulus, indicating more degree of renal apoptosis. The treatment of PTY-2r significantly reversed the immunoreactivity of all the protein as compared to DN rat.

### Effect of PTY-2r on urinary albumin excretion

**Fig. 5:**
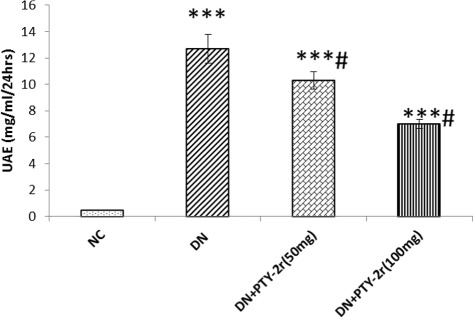
Effects of PTY-2r on urinary albumin excretion (UAE): NC, DN control, DN + PTY-2r (50 mg/100 g) and DN + PTY-2r (100 mg/100 g). Data are presented in mean ± SD (*n* = 6 in each group). ***p < 0.001 compared with group-1; #*p* < 0.001 compared with group-2

The urinary albumin excretion was measured, which showed a significant increase in DN rats compared with NC. The treatment with PTY-2r significantly reduced the albumin content in urine in a dose-dependent manner. The percent change in group-3 (50 mg) was 26.9% and 55%in group-4 (100 mg) (Fig. 5).

## Discussion

Several studies reported the direct involvement of oxidative stress in the pathogenesis of DN [2, 36, 37]. Excess generation of ROS leads to the severe cellular oxidative damage viz. LPO derivatives and this weaken the antioxidant machinery system (SOD, CAT, and GPx) [37, 38] in the kidney of DN rats. Kidney cells are more vulnerable to ROS mediated oxidative damage because of a high rate of oxygen consumptions [39]. Previously, we have reported that the STZ induced DN rats had clinical characteristics of increase proteinuria, urea and creatinine along with decreased creatinine clearance showed successful induction of DN and PTY-2r treatment significantly ameliorates these changes in a dose-dependent manner [22]. The present study is a continuous exploration of nephroprotective effect and underlying mechanism of PTY-2r in STZ induced DN rats. We observed the significant increase in oxidative stress markers i.e. ROS & LPO along with decreased ROS scavenging enzymes (SOD, CAT & GPx) in the kidney of DN rats. This is in accordance with the earlier reports, where inverse correlations between oxidative stress and activity of antioxidant enzymes have been reported [40]. The PTY-2r treatment significantly suppressed the increased production of ROS and LPO as compared to DN control rats and significantly raised the activity of antioxidant enzymes, in a dose-dependent manner, suggesting a significant antioxidant potential of PTY-2r. This antioxidant property of PTY-2r could be due to presence 2,3-dihydro-3,5-dihydroxy-6-methyl-4H-pyran-4-one (17.08%) and n- hexadecanoic acid which are identified through GC-MS analysis were reported as a strong antioxidant [41]. PTY-2r extract also rich in flavone, flavonoids, and polyphenols as shown in the phytochemical analysis of PTY-2r extract (Table 1). Thus, PTY-2r showed nephroprotective potential by reducing the oxidative stress through the restoration of antioxidant enzymes similar to other antioxidant agents like curcumin [42], resveratrol [43], berbarin [44] etc.

It has been reported that ROS-mediated oxidative stress also causes the disturbance in the balance between the pro-apoptotic (Bax) and antiapoptotic (Bcl-2) proteins, leading to an excess production of pro-apoptotic protein, which is susceptible to apoptosis [4]. The ratio of Bax and Bcl-2 protein is an important factor which determines cell survivability versus cell death [45]. The increased Bax/Bcl-2 ratio may cause damage to the mitochondrial membrane integrity which induces the cytochrome c (Cyt-c) release from the mitochondrial inner membrane (MIM). The leakage of Cyt-c from MIM may lead to activation of active Caspases-3 which may further activate caspase-9 [46]. Active Caspase-3 belongs to the cysteine proteases family and is involved in various forms of apoptotic pathways. It cleaves several substrates, notably DNA repairing enzymes viz. PARP [47]. Poly (ADP) ribose polymerase (PARP) is a nuclear enzyme responsible for ribosylation of nuclear enzymes and chromosomal proteins [48]. The formation of breaks in DNA strands during apoptosis is a stimulus of activation of the PARP-1. Thus PARP-1 plays a critical role in DNA repair and apoptosis. PARP-1 is a substrate for Caspases, particularly Caspase-3 [49]. Active Caspase-3 induced PARP-1 cleavage induces cellular disassembly, thus it acts as a specific marker for apoptosis [50]. Oxidative stress-induced apoptosis was evaluated by using the Bcl-2 to Bax ratio, cleaved PARP, and active Caspase-3 in the kidney of DN rats. Here, we found the diminished Bcl-2 expression and increased expression of Bax, active Caspase-3 and cleaved PARP-1 in the kidney of DN rats. However, the treatment with PTY-2r effectively ameliorated the changes in Bax and Bcl-2 family proteins and inhibited the cleavage of PARP-1 and activation of Caspase-3, showed its antiapoptotic potential. The antiapoptotic property of PTY-2r could be attributed to the presence of 5-Hydroxymethylfurfural, because in literature this compound is known for its antiapoptotic potential [51, 52]. Apoptosis of podocytes is the main contributor for albuminuria [53], although we have not checked, whether podocyte specifically dying but we are anticipating that podocyte death is because of significant changes in pro-apoptotic and antiapoptotic markers in the glomerular region of the kidney of DN rats. Thus, its prevention has been shown to reduce albumin excretion. Our results also showed that PTY-2r significantly decreased the urinary albumin excretion, which is a clinical characteristic of progression of DN. Previously, there are several reports stating that antioxidants have the ability to suppress the apoptosis [54, 55]. Cell apoptosis involves ROS as a critical intermediate messenger in its signalling cascade and antioxidants are responsible for scavenging of ROS thus inhibiting apoptosis [55]. This is also confirmed by our study that the antioxidant potential of PTY-2r is not only capable of suppressing oxidative stress but also inhibits the apoptotic markers in the kidney of DN rats.

## Conclusions

The PTY-2r significantly retards the progression of DN in diabetic rats by attenuating oxidative stress and apoptotic markers in the kidney of STZ-induced DN rats, thus reduced urinary albumin excretion. Hence, these findings strengthen the rationale for the therapeutic use of *Pueraria tuberosa* in the prevention of the diabetic nephropathy.

## Additional files

Additional file 1: **Table S1.** GCMS analysis showing chemical composition present in PTY-2r along with their structure and reported activity. Description of data- Data showing the compounds obtained from GCMS analysis and their reported activity in literature. (DOCX 280 kb)

## Abbreviations

CAT: Catalase
DN: Diabetic nephropathy
GPx: Glutathion peroxidase
LPO: Lipid peroxidation
ROS: Reactive oxygen species
SOD: Superoxide dismutase
STZ: Streptozotocin
