# Supplementary material for: Antioxidant and Antiapoptotic effect of aqueous extract of Pueraria tuberosa (Roxb. Ex Willd.) DC. On streptozotocin-induced diabetic nephropathy in rats
Source: BMC Complement Altern Med. 2018 May 11;18:156. doi: 10.1186/s12906-018-2221-x (PMC5948837; doi:10.1186/s12906-018-2221-x)
Supplement: Supplementary file 1 — Table S1. GCMS analysis showing chemical composition present in PTY-2r along with their structure and reported activity. Description of data- Data showing the compounds obtained from GCMS analysis and their reported activity in literature. (DOCX 280 kb) [file 12906_2018_2221_MOESM1_ESM.docx]

Table: S1 **GCMS Analysis Showing Chemical Composition Present in PTY-2r along with their Structure and Reported Activities**

| S.No. | R.Time | Name | Area | Area% | Formula | Mass  (g/mol) | Structure | Reported activity |
| --- | --- | --- | --- | --- | --- | --- | --- | --- |
| 1. | 4.225 | Gamma-Butyrolacton | 437557 | 0.32 | C_4_H_6_O_2_ | 86.09 |  | Antimicrobial activity [1] |
| 2. | 4.326 | 2-Methyl cyclopentanone | 385844 | 0.28 | C_6_H_10_O | 98.145 |  | No reported activity |
| 3. | 4.409 | 6-Oxa-bicyclo[3.1.0]hexan-3-one | 912069 | 0.66 | C_5_H_6_O_2_ | 98.101 |  | Antitrypanosoma  Activity [2], insecticidal activity [3] |
| 4. | 5.354 | 2,4-Dihydroxy-2,5-dimethyl-3(2H)-furan-3-one | 2449785 | 1.76 | C_6_H_8_O_4_ | 144.126 |  | No reported activity |
| 5. | 5.551 | 2H-Pyran-2,6(3H)-dione | 519708 | 0.37 | C_5_H_4_O_3_ | 112.084 |  | No reported activity |
| 6. | 6.439 | 1,4-Dimethylpiperazine | 1437808 | 1.04 | C_6_H_14_N_2_ | 114.192 |  | No reported activity |
| 7. | 6.528 | 4-Oxopentanoic acid | 610944 | 0.44 | C_5_H_8_O_3_ | 116.116 |  | Anti-inflammatory [4], biofuel [5] |
| 8. | 6.768 | 4-Hydroxy-2,5-dimethyl-3(2H)-furanone | 1359047 | 0.98 | C_6_H_8_O_3_ | 128.127 |  | Antibacterial, anti-microbial [6] flavor compound, Anti-allergic [7] |
| 9. | 7.164 | Cyclopentane, 1-acetyl-1,2-epoxy- | 1331050 | 0.96 | C_7_H_10_O_2_ | 126.155 |  | No reported activity |
| 10. | 7.339 | 5-Hydroxy-6-methyl-2,3-dihydro-4H-pyran-4-one | 252262 | 0.18 | C_6_H_8_O_3_ | 128.1259 |  | Potent aroma compound [8], |
| 11. | 7.851 | 2-Cyclopenten-1-One, 3-Ethyl-2-Hydroxy | 126131 | 0.09 | C_7_H_10_O_2_ | 126.155 |  | No reported activity |
| 12. | 8.334 | Acetic acid isobutyl ester | 1744745 | 1.26 |  |  |  | No reported activity |
| 13. | 8.623 | 2,3-dihydro-3,5-dihydroxy-6-methyl-4H-pyran-4-one | 23642509 | 17.03 | C_6_H_8_O_4_ | 144.126 |  | Flavonoid, Strong antioxidant, antifungal [9]. anti-inflammatory, anti-proliferative & pro-apoptotic[10] |
| 14. | 9.332 | 5-Hydroxy-2-hydroxymethyl-pyran-4-one | 462149 | 0.33 |  |  |  | Anti-inflammatory, anti-tumor, anti-microbial [11] |
| 15. | 10.26 | 5-Hydroxymethylfurfural | 24722467 | 17.80 | C_6_H_6_O_3_ | 126.111 |  | Anti-apoptotic [2,12] |
| 16. | 10.62 | Glycerol di-acetate | 8591188 | 6.19 | C_7_H_12_O_5_ | 176.168 |  | Insulin secretion[13] |
| 17. | 11.50 | Acetic acid, 3,4-dihydroxy-3-methyl-butyl ester | 3287648 | 2.37 | C_7_H_14_O_4_ | 162.185 |  | No reported activity |
| 18. | 11.95 | Cyclopentanone, 2-METHYL- | 1208940 | 0.87 | C_6_H_10_O | 98.145 |  | No reported activity |
| 19. | 15.09 | 2,5,5-Trimethyl-3-hexyn-2-ol | 940177 | 0.68 | C_9_H_16_O | 140.226 |  | No reported activity |
| 20. | 16.40 | Vanillic acid | 690483 | 0.50 | C_8_H_8_O_4_ | 168.148 |  | Antinociceptive [14], Antioxidant, Anti-inflammatory [15] |
| 21. | 17.90 | Caproic Acid | 3089295 | 2.22 | C_8_H_16_O_6_ | 208.209 |  | Antimicrobial, anti-fibrinolytic, Anti-bacterial and anti-inflammatory [16,17], treatment of menorrhagia [18] |
| 22. | 19.16 | 1,5-Anhydrohexitol | 6390956 | 4.60 | C_6_H_12_O_5_ | 164.157 |  | Anti-herpes [19], Anti-viral [20] |
| 23. | 19.78 | 4-O-Methylmannose | 6718536 | 4.84 | C_7_H_14_O_6_ | 194.183 g/mol |  | Antibacterial activity[21] |
| 24. | 20.16 | Quinic acid | 706027 | 0.51 | C_7_H_12_O_6_ | 192.167 g/mol |  | Antioxidant [22][23], antibacterial [24] anti-prostate cancer candidate [25] |
| 25. | 20.84 | Phytol, acetate | 592143 | 0.43 | C_22_H_42_O_2_ | 338.576 g/mol |  | Antioxidant, Anti-nociceptive [26], Antimycobacterial activity [27] |
| 26. | 21.46 | 10-Methyl-E-11-tridecen-1-ol propionate | 270804 | 0.20 | C_17_H_32_O_2_ | 268.441 g/mol |  | Antimicrobial activity |
| 27. | 22.63 | n-Hexadecanoic acid | 7188212 | 5.18 | C_16_H_32_O_2_ | 256.43 g/mol |  | Anti-inflammatory, antimicrobial [28], Bacteriocidal activity and antioxidant [2,29] |
| 28. | 23.06 | Stearic acid ethyl ester | 245405 | 0.18 | C_20_H_40_O_2_ | 312.538 g/mol |  | Antioxidant |
| 29. | 23.67 | 9-Octadecenoic acid (Z)- | 201065 | 6.69 | C_14_H_26_O_2_ | 226.36 g/mol |  | Anticancer, Anti-inflammatory, , 5-alpha  reductase inhibitor |
| 30. | 23.97 | Octadecanoic acid | 274621 | 0.20 | C_18_H_36_O_2_ | 284.484 g/mol |  | Anti-microbial , Antibacterial[29] Activity, Acaricidal [30] |
| 31. | 24.50 | octadeca-9,12-dienoyl chloride | 215100 | 0.15 | C_18_H_31_ClO | 298.895 g/mol |  | antituberculotic[31], Anit-inflammatory |
| 32. | 24.93 | 4-Tetradecyne | 1226165 | 0.88 | C_14_H_26_ | 194.362 g/mol |  | Antimicrobial [32] |
| 33. | 25.38 | Z- 7-Tetradecenal | 884788 | 0.64 | C_14_H_26_O | 210.361 g/mol |  | sex pheromone[33] |
| 34. | 29.82 | Hexadecanoic acid, 2-hydroxy-1-(hydroxymethyl)ethyl ester | 10747400 | 7.74 | C_19_H_38_O_4_ | 330.509 g/mol |  | Hemolytic, pesticide, flavour,  Antioxidant [34] |
| 35. | 30.87 | Leinoleic acid | 3571071 | 2.57 | C_18_H_32_O_2_ | 280.452 |  | Anti-tubercular[35], antioxidant [36], anti-inflammatory [37] |
| 36. | 32.25 | 2,5-Methano-1H-Inden-7(4H)-one, hexahydro- | 4652794 | 3.35 | C_10_H_14_O | 150.221 |  | No reported activity |
| 37. | 32.47 | Octadecanoic acid, 2,3-dihydroxypropyl ester | 5686836 | 4.10 | C_21_H_42_O_4_ | 358.563 |  | No reported activity |

References

1. Mehetre D, Ghadge S, Chabukswar A, Lokhande P. Synthesis and Evaluation of Antimicrobial activity of Gamma Butyrolactone. Int. J. Pharm. Phytopharm. Res. 2013;2:412–4.

2. Hadi Hameed I, - J, Al MJ, Jihadi Mohammed G. Anti-bacterial, Antifungal Activity and Chemical Analysis of Punica grantanum (Pomegranate peel) Using GC-MS and FTIR Spectroscopy * 3. Int. J. Pharmacogn. Phytochem. Res. 2016;8:480–94.

3. De Alvarenga ES, Carneiro VMT, Resende GC, Picanço MC, De E, Farias S, et al. Synthesis and Insecticidal Activity of an Oxabicyclolactone and Novel Pyrethroids. Molecules. 2012;17:13989–4001.

4. Fujino M, Nishio Y, Ito H, Tanaka T, Li X-K. 5-Aminolevulinic acid regulates the inflammatory response and alloimmune reaction. Int. Immunopharmacol. J. 2016;37:71–8.

5. Yan K, Chen A. Selective hydrogenation of furfural and levulinic acid to biofuels on the ecofriendly Cu-Fe catalyst. Fuel. 2014;115:101–8.

6. Sung WS, Jung HJ, Park K, Kim HS, Lee I-S, Lee DG. 2,5-dimethyl-4-hydroxy-3(2H)-furanone (DMHF); antimicrobial compound with cell cycle arrest in nosocomial pathogens. Life Sci. 2007;80:586–91.

7. Mi H, Hiramoto K, Kujirai K, Ando K, Ikarashi Y, Kikugawa K. Effect of food reductones, 2,5-dimethyl-4-hydroxy-3(2H)-furanone (DMHF) and hydroxyhydroquinone (HHQ), on lipid peroxidation and type IV and I allergy responses of mouse. J. Agric. Food Chem. 2001;49:4950–5.

8. Preininger M, Gimelfarb L, Li HC, Dias BE, Fahmy F, White J. Identification of dihydromaltol (2,3-dihydro-5-hydroxy6-methyl-4h-pyran-4- one) in ryazhenka kefir and comparative sensory impact assessment of related cycloenolones. J. Agric. Food Chem. 2009;57:9902–8.

9. Yu X, Zhao M, Liu F, Zeng S, Hu J. Identification of 2,3-dihydro-3,5-dihydroxy-6-methyl-4H-pyran-4-one as a strong antioxidant in glucose–histidine Maillard reaction products. Food Res. Int. 2013;51:397–403.

10. Ban JO, Hwang IG, Kim TM, Hwang BY, Lee US, Jeong H-S, et al. Anti-proliferate and pro-apoptotic effects of 2,3-dihydro-3,5-dihydroxy-6-methyl-4H-pyranone through inactivation of NF-κB in Human Colon Cancer Cells. Arch. Pharm. Res. 2007;30:1455–63.

11. Liu X, Xia W, Jiang Q, Xu Y, Yu P. Synthesis, Characterization, and Antimicrobial Activity of Kojic Acid Grafted Chitosan Oligosaccharide. J. Agric. Food Chem. 2014;62:297–303.

12. Gu H, Jiang Z, Wang M, Jiang H, Zhao F, Ding X, et al. 5-Hydroxymethylfurfural from wine-processed Fructus corni inhibits hippocampal neuron apoptosis. Neural Regen. Res. 2013;8:2605–14.

13. Govindappa M, Channabasava GM, Cp C, Ts S. QR Code for Mobile users In Vitro Antidiabetic Activity of Three Fractions of Methanol Extracts of Loranthus Micranthus, Identification of Phytoconstituents by GC-MS and Possible Mechanism Identified by GEMDOCK Method. Asian J. Biomed. Pharm. Sci. 2014;4:34–41.

14. Yrbas M de los A, Morucci F, Alonso R, Gorzalczany S. Pharmacological mechanism underlying the antinociceptive activity of vanillic acid. Pharmacol. Biochem. Behav. 2015;132:88–95.

15. Calixto-Campos C, Carvalho TT, Hohmann MSN, Pinho-Ribeiro FA, Fattori V, Manchope MF, et al. Vanillic Acid Inhibits Inflammatory Pain by Inhibiting Neutrophil Recruitment, Oxidative Stress, Cytokine Production, and NFκB Activation in Mice. J. Nat. Prod. 2015;78:1799–808.

16. HOPKINS TF, MEITES J. Effects of epsilon-amino-caproic acid on prolactin-inactivating and fibrinolytic activities of streptokinase-activated plasminogen. Proc. Soc. Exp. Biol. Med. 1963;112:830–2.

17. Marounek M, Putthana V, Benada O, Lukešová D. Antimicrobial Activities of Medium-chain Fatty Acids and Monoacylglycerols on Cronobacter sakazakii DBM 3157 T and Cronobacter malonaticus DBM 3148. Czech J. Food Sci. 2012;30:573–80.

18. Mehta BC, Parekh DS. epsilon-amino-caproic acid in the treatment of menorrhagia. J. Postgrad. Med. 1977;23:121–3.

19. Verheggen I, Van Aerschot A, Toppet S, Snoeck R, Janssen G, Balzarini J, et al. Synthesis and antiherpes virus activity of 1,5-anhydrohexitol nucleosides. J. Med. Chem. 1993;36:2033–40.

20. Hossain N, Rozenski J, De Clercq E, Herdewijn P. Synthesis and Antiviral Activity of the alpha-Analogues of 1,5-Anhydrohexitol Nucleosides (1,5-Anhydro-2,3-dideoxy-D-ribohexitol Nucleosides). J. Org. Chem. 1997;62:2442–7.

21. Zab R, Kumar A, Bhaskar A. Phytochemical evaluation by GC-MS and in vitro antioxidant activity of Punica granatum fruit rind extract. J. Chem. Pharm. Res. 2012;4:2869–73.

22. Pero RW, Lund H, Leanderson T. Antioxidant metabolism induced by quinic acid. increased urinary excretion of tryptophan and nicotinamide. Phyther. Res. 2009;23:335–46.

23. Hung TM, Na M, Thuong PT, Su ND, Sok D, Song KS, et al. Antioxidant activity of caffeoyl quinic acid derivatives from the roots of Dipsacus asper Wall. J. Ethnopharmacol. 2006;108:188–92.

24. Gohari AR, Saeidnia S, Mollazadeh K, Yassa N, Malmir M, Shahverdi AR. Isolation of a new quinic acid derivative and its antibacterial modulating activity. Daru. 2010;18:69–73.

25. Padmini E, Inbathamizh L. Quinic Acid As A Potent Drug Candidate For Prostrate Cancer – A Comparative Pharmacokinetics Approch. Asian J. Pharm. Clin. Res. 2017;6:106–12.

26. Santos CC de MP, Salvadori MS, Mota VG, Costa LM, de Almeida AAC, de Oliveira GAL, et al. Antinociceptive and Antioxidant Activities of Phytol In Vivo and In Vitro Models. Neurosci. J. 2013;2013:1–9.

27. Rajab M, Cantrell C, Franzblau S, Fischer N. Antimycobacterial Activity of ( E )-Phytol and Derivatives: A Preliminary Structure-Activity Study. Planta Med. 1998;64:2–4.

28. Aparna V, Dileep K V., Mandal PK, Karthe P, Sadasivan C, Haridas M. Anti-Inflammatory Property of n-Hexadecanoic Acid: Structural Evidence and Kinetic Assessment. Chem. Biol. Drug Des. 2012;80:434–9.

29. Zhong-Hui P, Zhang Y-Q, Zhong-Qiong Y, Jiao X, Ren-Yong J, Yang L, et al. Antibacterial Activity of 9-Octadecanoic Acid-Hexadecanoic Acid- Tetrahydrofuran-3,4-Diyl Ester from Neem Oil. Agric. Sci. China. 2010;9:1236–40.

30. Du Y-H, Li J-L, Jia R-Y, Yin Z-Q, Li X-T, Lv C, et al. Acaricidal activity of four fractions and octadecanoic acid-tetrahydrofuran-3,4-diyl ester isolated from chloroform extracts of neem (Azadirachta indica) oil against Sarcoptes scabiei var. cuniculi larvae in vitro. Vet. Parasitol. 2009;163:175–8.

31. Hussain A, Rather MA, Shah AM, Bhat ZS, Shah A, Ahmad Z, et al. Antituberculotic activity of actinobacteria isolated from the rare habitats. Lett. Appl. Microbiol. 2017;65:256–64.

32. Ara I, Shinwari MMA, Rashed SA, Bakir MA. Evaluation of Antimicrobial Properties of Two Different Extracts of Juglans regia Tree Bark and Search for Their Compounds Using Gas Chromatohraphy-Mass Spectrum. Int. J. Biol. 2013;5:92.

33. Steck WF, Underhill EW, Bailey BK, Chisholm MD. ( Z )-7-Tetradecenal, a Seasonally Dependent Sex Pheromone of the w-Marked Cutworm, Spaelotis clandestina (Harris) (Lepidoptera: Noctuidae) 1. Environ. Entomol. 1982;11:1119–22.

34. Tyagi T, Agarwal M. Phytochemical screening and GC-MS analysis of bioactive constituents in the ethanolic extract of Pistia stratiotes L. and Eichhornia crassipes (Mart.) solms. J. Pharmacogn. Phytochem. JPP. 2017;6:195–206.

35. Won Hyung Choi. Evaluation of anti-tubercular activity of linolenic acid and conjugated-linoleic acid as effective inhibitors against Mycobacterium tuberculosis. Asian Pac. J. Trop. Med. 2016;9:125–9.

36. Fagali N, Catalá A. Antioxidant activity of conjugated linoleic acid isomers, linoleic acid and its methyl ester determined by photoemission and DPPH techniques. Biophys. Chem. 2008;137:56–62.

37. Anand R, Kaithwas G. Anti-inflammatory Potential of Alpha-Linolenic Acid Mediated Through Selective COX Inhibition: Computational and Experimental Data. Inflammation. 2014;37:1297–306.
